# Comparative analysis of stress levels and coping strategies in parents of neurodivergent and neurotypical children

**DOI:** 10.3389/frcha.2025.1619993

**Published:** 2025-08-22

**Authors:** Lessa A. Méndez-Lara, Rodrigo Ramirez-Rodriguez, Edgar Santos, Angel Puig-Lagunes

**Affiliations:** ^1^ Faculty of Medicine, Veracruzana University, Minatitlan, Mexico; ^2^ Autonomous University of Mexico City, Mexico City, Mexico; ^3^ Department of Neurosurgery, University of Oldenburg, Oldenburg, Germany

**Keywords:** autism spectrum disorder, attention deficit disorder with hyperactivity, behavioral mechanisms, coping skills, parents

## Abstract

Parents of children with autism spectrum disorder (ASD) and attention deficit hyperactivity disorder (ADHD) face unique challenges that may significantly increase stress levels, potentially impacting the emotional well-being of the entire family. In Mexico, limited research has examined the association between parental stress and coping strategies among families with children with developmental disabilities. This study aimed to compare stress levels and coping strategies among parents of children with ASD, ADHD, and neurotypical developing (NTD) children, as well as to analyze differences in coping styles across these groups. A cross-sectional, descriptive-comparative design was employed with 212 parents of children aged 3 to 5 years, with a formal clinical diagnosis made by a pediatric neurologist. Participants completed validated questionnaires assessing parental stress and coping styles. Results revealed that parents of children with ASD and ADHD reported significantly higher stress levels (*M* = 116.7 and *M* = 88.1, respectively) compared to parents of NTD children (*M* = 67.2). Significant differences in coping strategies were observed (*p* < .001); 100% of ASD/ADHD parents used emotion-focused coping, whereas 94.93% of NTD parents used problem-focused coping. These findings emphasize the importance of designing interventions to support coping and stress regulation in parents of children with developmental disorders.

## Introduction

Recent years have seen a marked increase in parental stress, attributable to a combination of social, economic, and cultural shifts. A study published in the Proceedings of the National Academy of Sciences demonstrates that global trends in emotional distress highlight this increase, rising from 25.16% in 2009 to 31.19% in 2021, representing a 6.03 percentage point increase over the 12 years (1). Furthermore, the American Psychological Association (APA) reports that 70% of parents have indicated a substantial escalation in stress levels during the period of the pandemic (2). This rise in stress levels has been attributed to the increased parental responsibilities that accompany managing remote learning and balancing work and childcare obligations.

Parenting during early childhood (ages 3–5) is a particularly sensitive period, marked by the growing demands associated with children's rapid cognitive, emotional, and behavioral development. This stage is foundational for shaping communication patterns, fostering emotional bonds, and supporting long-term socioemotional outcomes. While all parents face challenges during this developmental window, those raising children with neurodevelopmental conditions—such as autism spectrum disorder (ASD) and attention deficit hyperactivity disorder (ADHD)—are often confronted with additional complexities, including diagnostic ambiguity, demanding treatment decisions, and heightened caregiving responsibilities (3).

These increased demands are not limited to the child's condition but are also shaped by the psychological, social and economic context in which parenting takes place. Research has shown that the early stages of caregiving significantly influence parental stress and the establishment of coping mechanisms (4–7). Particularly, in families of children with ASD and ADHD, parental well-being is affected by the presence of co-occurring behavioral and mental health issues, such as self-injury, aggression, impulsivity, and noncompliance (8, 9).

Moreover, parents' subjective experiences—including how they perceive their child's behavior, internalized stigma, and financial strain—can exacerbate stress levels and influence their responsiveness to interventions. Parents who perceive their child's behavior as more problematic often report higher emotional distress, whichmay hinder their engagement in supportive programs (5, 8, 10). In contrast, parents of children with typical neurodevelopment (NTD) generally report lower levels of stress, likely due to fewer behavioral disruptions and more manageable caregiving demands (7, 11, 12).

The coping strategies employed by parents are critical to managing stress and maintaining family well-being. Positive or problem-focused strategies, such as problem-solving and seeking social support, have been shown to promote emotional attachment and positive self-esteem, enhancing family cohesion and adaptability (13, 14). In contrast, negative or emotion-focused strategies, such as avoidance, self-blame, and wishful thinking, have been shown to hinder parents' ability to regulate family functioning and respond effectively to caregiving demands (15, 16). Recent studies have shown that poor psychological well-being among caregivers, including increased parenting stress, is associated with a decrease in the quality of parent-child relationships, academic performance, and social integration (13, 17). Conversely, a strong parent-child bond has been associated with positive family outcomes, such as reduced emotional and behavioral dysregulation in children with ASD (15, 16, 18).

Despite the plethora of studies that have examined parental stress in families of children with developmental disabilities, only a limited number have specifically examined the direct relationship between parents' coping strategies and the levels of stress they experience. It is imperative to understand these dynamics to identify specific stress profiles and develop tailored interventions to promote the well-being of parents and family units.

Moreover, in the Mexican context, such an analysis could shed light on disparities in access to resources and support for families of children with ASD and ADHD. Parenting a child with a neurodevelopmental condition in Mexico presents unique challenges shaped by a complex interplay of social, economic, and healthcare-related factors. Limited access to specialized services, regional disparities in diagnostic and therapeutic resources, and persistent stigma surrounding mental health can significantly intensify parental stress and hinder the development of effective coping strategies. Moreover, sociocultural expectations regarding parenting roles—often disproportionately placed on mothers—may further exacerbate emotional and caregiving burdens (19, 20).

In comparison to high-income countries, where more robust support systems may be in place, parents in Mexico often rely on informal networks or face systemic barriers to care. These contextual differences underline the importance of examining parental stress and coping in settings where structural limitations may alter both the intensity and nature of these experiences. Framing the study within this national context may offer valuable insights for cross-cultural comparisons and contribute to the global understanding of parenting in neurodiversity (19, 20).

This study aimed to compare stress levels and coping strategies among parents of children with ASD, ADHD, and NTD, as well as to analyze differences in coping styles across these groups. As part of an exploratory component of the study, a principal component analysis (PCA) was conducted to examine whether underlying patterns in parental stress dimensions and coping strategies could reveal meaningful group-level distinctions, particularly between parents of autistic children and those with ADHD. While PCA is not used to test specific hypotheses, it is a valuable tool for dimensionality reduction and visualizing latent structures in complex psychosocial data (21). In this context, the PCA served to complement our inferential analyses by offering a visual and multivariate perspective on how stress-related responses may differ across diagnostic groups. Findings from such studies can inform the design of national programs aimed at reducing parental stress and promoting positive coping strategies, thereby contributing to healthier and more balanced family development.

## Materials and methods

### Procedure

A cross-sectional, descriptive-comparative, and quantitative study was conducted from October to November 2024 in Minatitlan, Veracruz, Mexico. The study focused on parents of children aged 3–5 years, a developmental stage characterized by rapid changes in communication, behavior, and autonomy. The early years were selected due to their critical role in shaping parent–child dynamics and because early parenting demands can be particularly taxing for families managing neurodevelopmental conditions. By focusing on this age group, the study aimed to identify the unique stressors and coping patterns associated with early caregiving in individuals with different neurotypes (4–7).

Parents were recruited using a non-probability convenience sampling method. Participants were approached in three educational and care centers where they routinely brought their children for services: *Centro de Atención Múltiple No. 7* and *Centro de Atención Neuropsicológica y de Lenguaje* (by parents of children with ASD or ADHD, diagnosis), and public kindergarten María Enriqueta Camarillo (parents of children with NTD). Recruitment was conducted in person, following prior authorization from the participating institutions. Parents were informed about the study's objectives and procedures and were invited to participate voluntarily. Data collection was carried out through a Google Forms survey, with trained research personnel providing personalized assistance during its administration. This support ensured proper understanding of the questions, clarification of any doubts, and accurate completion of the instruments.

### Inclusion and exclusion criteria

Parents or legal guardians of children aged 3–5 years with a formal diagnosis of autism spectrum disorder (ASD)or attention-deficit/hyperactivity disorder (ADHD), with or without comorbidities, were eligible to participate. The diagnosis must have been made at least one year before the study by a certified pediatric neurologist and may include parents of children receiving pharmacological or non-pharmacological treatment.

Participants were excluded if the child presented comorbid complex genetic conditions, such as Rett syndrome, Fragile X syndrome, or other syndromes known to independently affect developmental, behavioral, or emotional variables relevant to the study. Additionally, parents were excluded if they had a clinically diagnosed and untreated psychiatric condition likely to interfere with their ability to provide reliable responses, such as schizophrenia, untreated bipolar disorder, or non-stabilized major depressive episodes. Only one parent or primary caregiver per child was invited to complete the questionnaires. Participation of both parents was not required; instead, the responding parent was the one most actively involved in the child's day-to-day care. Parents of children with NTD in the same age range were also included using the same in-person recruitment procedures as for children with ASD or ADHD. All participants were required to sign an informed consent form and complete the questionnaires in full.

One participant was excluded from the final analysis due to incomplete responses, which prevented accurate scoring across the study instruments. This exclusion was made to maintain data integrity and ensure the validityof statistical comparisons among groups.

#### Sociodemographic data

The questionnaire encompassed a wide range of demographic and clinical information, including relationship status, number of children, child's diagnosis, time since diagnosis, child's age, treatment modalities (behavioral and/or pharmacological), duration of treatment, parent's age, marital status, education level, current employment status, and socioeconomic status which was categorized based on monthly household income in Mexican pesos, following general thresholds reported by the National Institute of Statistics and Geography (INEGI). A low socioeconomic level was defined as earning between $0 and $11,000 MXN per month (approximately $0 to USD 647), a medium level as earning between $11,000 and $22,000 MXN (roughly $647 to USD 1,294), and a high socioeconomic level as earning between $22,000 and $77,000 MXN (approximately $1,294 to USD 4,529). These ranges are consistent with national socioeconomic classifications in Mexico, where middle-class income typically begins at approximately $22,000 MXN per month (22).

#### Parental stress index-short form (PSI-SF

The scale was developed by Dr. Richard R. Abidin as a tool to assess parental stress as perceived by parents in their parental role (23). The abridged version, designated as the PSI-SF, comprises 36 items that address three domains of parental stress: parental distress (*α* = 0.87), dysfunctional parent-child interaction (*α* = 0.80), and a difficult child (*α* = 0.85). Each item is evaluated by parents on a 5-point Likert scale, and a total parental stress score is derived by summing all responses, with higher scores indicating greater parental stress.

Scores ranging from 15 to 80 are classified as usual, while those exceeding 85 are deemed to be clinically significant and necessitate further observation (24, 25). In this study, the PSI-SF demonstrated adequate psychometric properties for parental distress (*α* = 0.70), difficult child (*α* = 0.88), and a marginal level of acceptability for dysfunctional parent-child interaction (*α* = 0.67). In our study, scale reliability was determined as follows: Cronbach's alpha was determined to be 0.898, and McDonald's omega was found to be 0.901. The questionnaire was administered in Spanish, using the validated version of the PSI-SF for Spanish-speaking populations (26). Parental stress levels were categorized based on raw scores obtained from the PSI-SF. A score below 59 was interpreted as indicating low stress, while scores ranging from 61 to 82 were considered reflective of everyday or adequate stress. Scores equal to or greater than 86 were classified as high stress, and scores equal to or above 91 were deemed clinically significant, suggesting the need for further evaluation or intervention.

#### Coping styles questionnaire (CSQ)

In this study, the Coping Strategies Questionnaire (CSQ) was used to assess parental coping styles. The CSQ is a Spanish-language adaptation of the *Ways of Coping Checklist-Revised* (WCC-R) initially developed by Vitaliano et al. (27) and adapted by Flórez-Alarcón (28) for Spanish-speaking populations. This instrument was developed within the framework of Lazarus and Folkman's (29) transactional theory of stress, which distinguishes between two primary coping styles: problem-focused coping, involving active efforts to manage or alter the source of stress, and emotion-focused coping, which aims to regulate the emotional stress response.

The CSQ comprises 42 items rated on a four-point Likert scale, ranging from “never” to “frequently,” and is divided into five subscales reflecting distinct coping strategies. Two of these subscales—problem-solving (15 items) and seeking social support (6 items)—represent problem-focused or positive coping strategies. The remaining three subscales—avoidance (10 items), self-blame (3 items), and wishful thinking (8 items)—are categorized as emotion-focused or negative coping strategies. Problem-solving involves direct actions to confront the stressor, such as planning or gathering information; seeking social support refers to mobilizing external assistance; avoidance entails behavioral or cognitive disengagement; self-blame reflects internal attribution of responsibility; and wishful thinking encompasses passive hopes for change without active steps toward resolution (30, 31).

The original validation study by Flórez-Alarcón (28) demonstrated high internal consistency (Cronbach's *α* = 0.87) and scale homogeneity, supporting the psychometric soundness of the instrument. In our study, scale reliability was determined as follows: Cronbach's alpha was determined to be 0.901, and McDonald's omega was found to be 0.906.

To identify the predominant coping style used by participants, the relative scoring method proposed by Vitaliano et al. (27) was applied. This method involves calculating the mean score of each subscale and dividing it by the sum of the means of all five subscales, thereby obtaining a proportional score that reflects the relative use of each coping strategy. Drawing on Lazarus and Folkman's (29) model, Flórez-Alarcón (28) suggested that an adaptive coping profile consists of approximately 70% problem-focused coping and 30% emotion-focused coping. These proportions have been supported by subsequent authors (30–32) as indicative of a functional and adaptive response to stress, particularly in contexts where some stressors can be modified or managed effectively. Conversely, substantial deviations from this ratio—such as a predominant reliance on avoidance or self-blame—may signal maladaptive coping processes.

### Statistical analysis

The association between the domain scores of PSI-SF and CSQ was assessed using Spearman's correlation coefficient. The strength of the association was then categorized as follows: weak (0.30–0.50), moderate (0.50–0.70), strong (0.70–0.90), and very strong (0.90–1.00) (33). To further explore the underlying structure of the data and reduce dimensionality, Principal Component Analysis (PCA) was employed. PCA is an unsupervised dimensionality reduction technique that transforms a set of potentially correlated variables into a smaller number of uncorrelated components, called principal components. These components are constructed such that the first principal component captures the most significant amount of variance in the data, with each subsequent component accounting for the highest remaining variance while being orthogonal (i.e., statistically independent or uncorrelated) to the previous ones. By maximizing the variance, PCA enables the identification of underlying patterns and structures in complex multivariate datasets. In the context of this study, PCA was applied to the domain scores of the PSI-SF and CSQ to uncover the dimensions that most strongly differentiate between parents of children with NTD, ADHD, or ASD. Clusters were defined using a data-driven approach based on the results of PCA. This approach facilitates a clearer understanding of which domains are most representative or informative in distinguishing stress and coping profiles across these diagnostic groups.

The PSI-SF or CSQ domains were analyzed using one-way ANOVA or Welch's ANOVA to evaluate differences between parent groups. The magnitude of the detected differences was estimated using Omegasquared (*ω*^2^). Values below 0.01 were considered very small, between 0.01 and 0.06 were considered small, 0.06–0.14 were considered medium, and greater than 0.14 were considered significant (34). Considering the characteristics of the sample and the assumptions met for the omnibus tests, the Games-Howell test was used to conduct multiple comparisons (35). For categorical variables, the dependence of sociodemographic data or coping style between parent groups, as well as the relationship between parental stress and coping style, was assessed using either the Chi-square test or Fisher's Exact test. The Cramér's *V* was calculated as an effect size for categorical variables, with the following interpretations: >0 (no or very weak), >0.05 (weak), >0.10 (moderate), >0.15 (strong), and >0.25 (very strong) (36). Finally, binomial logistic regression was used to predict the probabilities of coping style in response to parental stress, with the emotion-focused style (code = 1) serving as the reference level. Data analyses and visualizations were carried out using the JupyterLab interface within the Anaconda distribution of Python and the RStudio integrated development environment for macOS.

### Ethics

This research was approved by the Institutional Research and Ethics Committee (FOLIO: CI-001-2024). In addition, the research complied with the General Health Law, articles 13, 14, 16, 20 and 36, as well as the tenets the Declaration of Helsinki and the General Health Law of Mexico, chapters 96, 100 and 102 (37, 38).

## Results

### Sociodemographic characteristics

A total of 212 parents participated in the study, with 211 returning fully complete questionnaires. Consequently, the final sample comprised 211 participants, reflecting a response rate of 99.5% after excluding one case due to incomplete data. The sample included 37.4% parents of children with ASD (*n* = 79), 30.8% with ADHD (*n* = 65), and 31.7% with NTD (*n* = 67). Group differences were observed across several sociodemographic and treatment-related variables (Table 1).

**Table 1:** Frequencies and percentages of sociodemographic information for parents with children with NTD, ADHD, and ASD.

Variable	NTD (*n* = 79)	ADHD (*n* = 65)	ASD (*n* = 67)	ES (Cramer's *V*)
Parent's age				0.54
21–30	24 (15%)	65 (42%)	67 (43%)	
31–40	40 (100%)	0 (0%)	(0%)	
>41	15 (100%)	0 (0%)	(0%)	
Respondent's kinship				—
Mother	70 (37%)	56 (30%)	63 (33%)	
Father	9 (41%)	9 (41%)	4 (18%)	
Respondent's marital status				—
Single	20 (34%)	23 (39%)	16 (27%)	
Married	59 (39%)	42 (28%)	51 (33%)	
Respondent's educational level				0.22
High school or lower	60 (50%)	26 (22%)	33 (28%)	
Bachelor's degree	15 (20%)	32 (42%)	29 (38%)	
Postgraduate degree	4 (25%)	7 (44%)	5 (31%)	
Current employment status				—
(Employed)				
Yes	44 (37%)	42 (35%)	34 (28%)	
No	35 (38%)	23 (25%)	33 (37%)	
Socioeconomic level				—
Low (0–647 USD)	32 (40.50%)	28 (43.07%)	33 (49.25%)	
Medium (>647–1,294 USD)	37 (46.83%)	30 (46.15%)	29 (43.28%)	
High (>1,294–4,529 USD)	10 (12.65%)	7 (10.76%)	5 (7.46%)	
No. children				0.16
1	26 (27%)	35 (37%)	34 (36%)	
2	47 (50%)	21 (22%)	26 (28%)	
3	6 (27%)	9 (41%)	7 (32%)	
Child's age				—
2	1 (50%)	0 (%)	1 (50%)	
3	7 (44%)	5 (31%)	4 (25%)	
4	24 (44%)	11 (20%)	20 (36%)	
5	47 (34%)	49 (36%)	42 (30%)	
Behavioral treatment				0.77
Yes	2 (2%)	55 (50%)	54 (48%)	
No	77 (77%)	10 (10%)	13 (13%)	
Pharmacological treatment				0.77
Yes	2 (2%)	35 (41%)	48 (57%)	
No	77 (61%)	30 (24%)	19 (15%)	

ES, effect size.

It was observed that a 100% prevalence of clinically relevant levels of stress was experienced by parents of children diagnosed with ASD. Conversely, all parents of children with ADHD reported a high degree of stress, and parents of children with NTD exhibited a functional level of stress. The study revealed that 100% of parents of children with ASD and ADHD reported utilizing emotion-focused coping strategies, while 94.93% of parents of children with NTD primarily employed problem-focused coping strategies.

### Correlation

**Figure 1:**
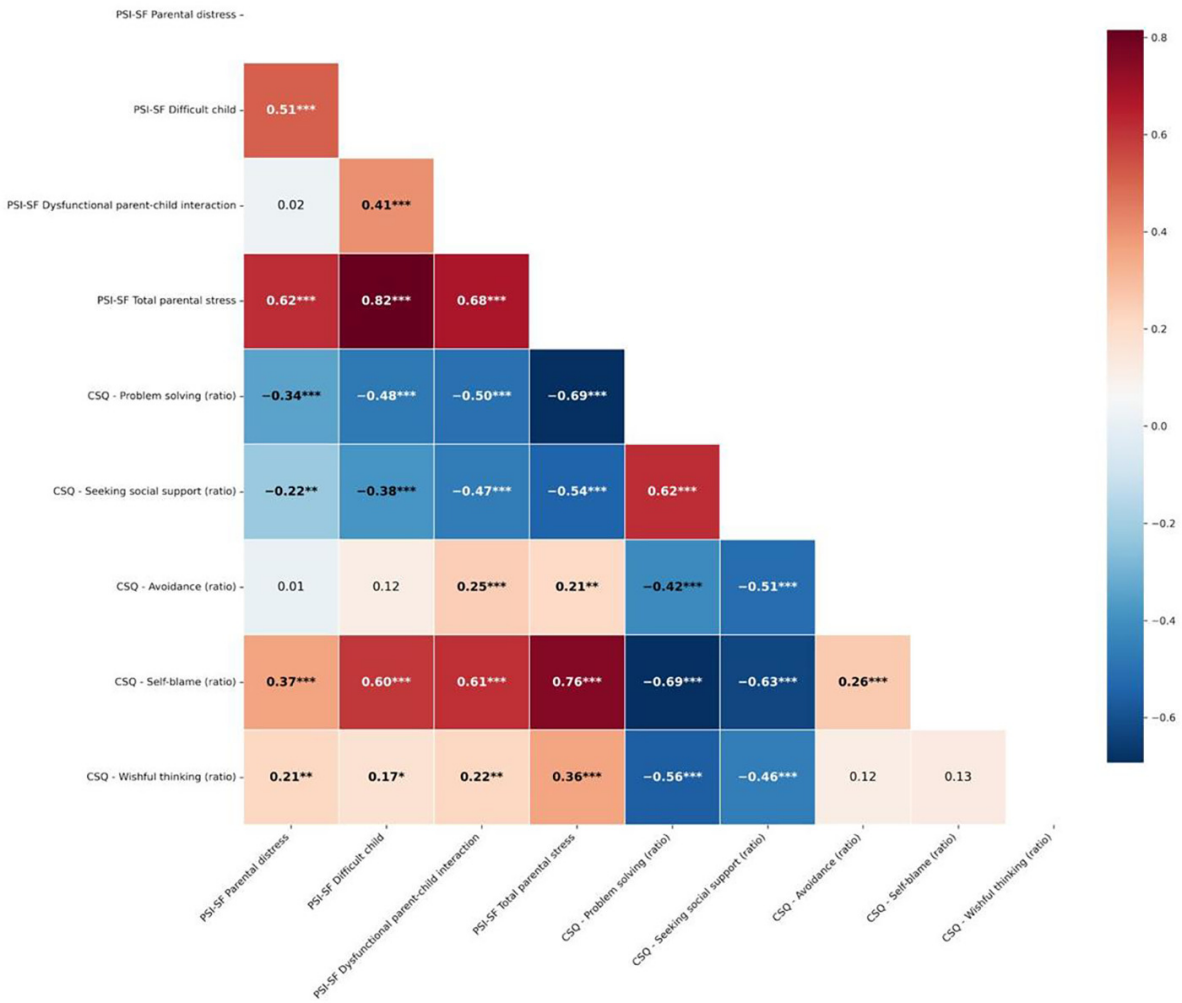
Matrix correlation of spearman correlation between PSI-SF and CSQ. Ratios reflect the proportion of each coping strategy, calculated by dividing the mean of each subscale by the sum of all five subscale means. Asterisks denote significant difference: *p < 0.05; **p < 0.01; ***p < 0.001.

Significant correlations were observed between parental stress dimensions and coping strategies (Figure 1). Total parental stress was strongly and positively associated with self-blame (*r* = 0.76, *p* < 0.001), perception of a difficult child (*r* = 0.82, *p* < 0.001), and dysfunctional parent–child interactions (r = 0.68, *p* < 0.001). Adaptive coping strategies, such as seeking social support (*r* = –.54, *p* < .001) and problem-solving (*r* = –0.69, *p* < 0.001), were negatively associated with total parental stress. In contrast, maladaptive strategies, including self-blame (*r* = 0.76, *p* < 0.001) and wishful thinking (*r* = 0.36, *p* < 0.001), were positively associated with higher stress levels. Overall, adaptive coping strategies tended to correlate negatively with various components of parental stress. In contrast, maladaptive strategies were positively associated, highlighting the potential buffering role—that is, these strategies may help mitigate or reduce the impact of stressors on parents' psychological well-being. In contrast, maladaptive strategies were positively associated with stress, potentially exacerbating its effects.

### Principal component analysis

**Figure 2:**
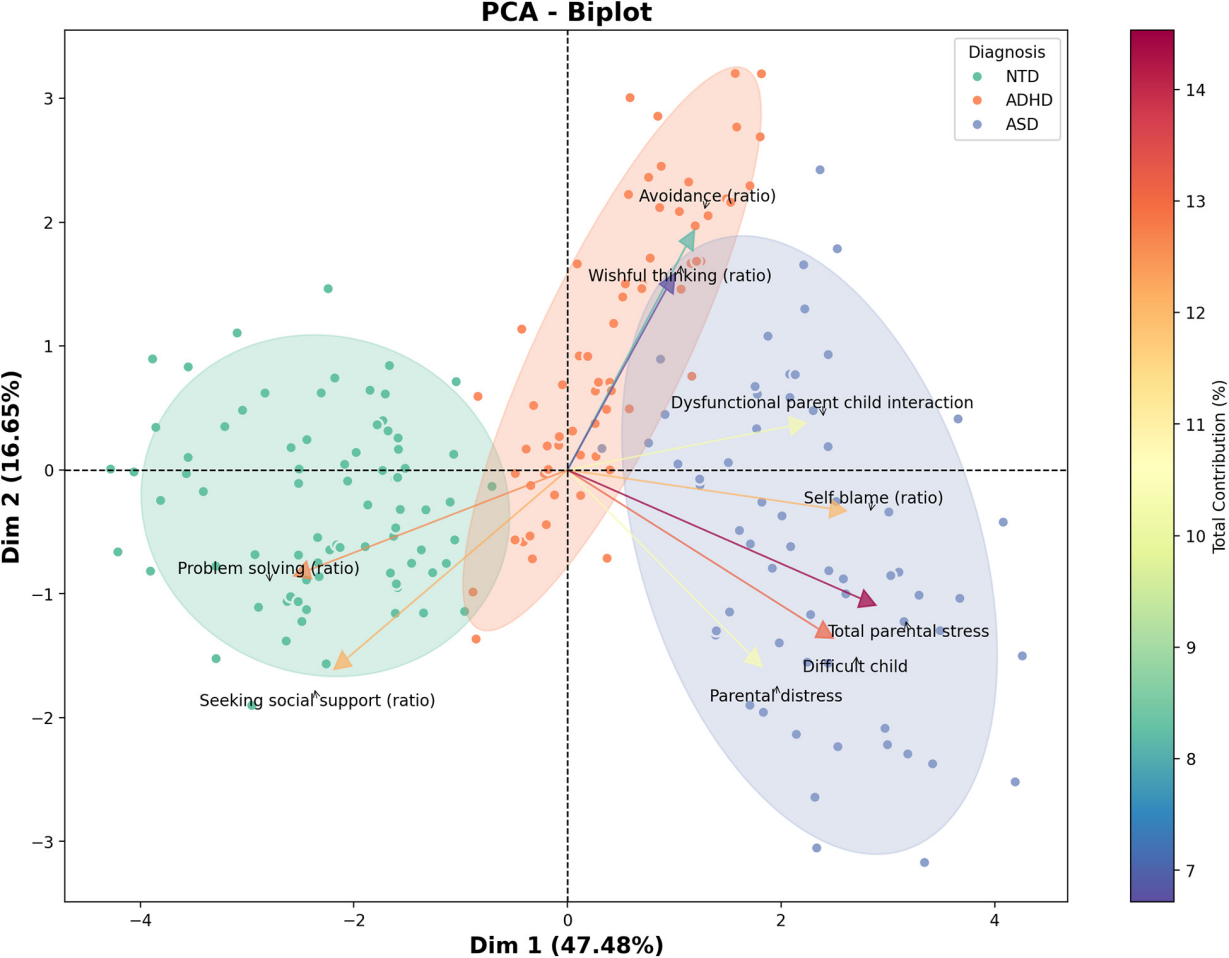
Patterns of maximum variance for parents of children with NTD, ADHD, and ASD. The length of the rows denotes the degree of variance of each dependent variable within each cluster. The first principal component (Dimension 1), which explains 47.48% of the total variance, primarily captures overall parental stress and coping difficulties across groups. The second component (Dimension 2), accounting for 16.55% of the variance, distinguishes parents based on patterns of perceived social support and problem-solving strategies. Together, these components account for 64.93% of the total variance, allowing a meaningful visualization of the main patterns differentiating the clusters. Some variables are expressed as ratios, calculated by dividing the mean score of each subscale by the sum of the means of all five subscales. This method reflects the proportional use of each coping strategy rather than raw or summed values.

The identification of patterns of maximum variance across three clusters, along with the contributions of their respective variables, is illustrated in Figure 2. Parents of children with NTD were primarily characterized by their problem-solving abilities and tendency to seek social support. Conversely, parents of children with ADHD exhibited a proclivity for avoidance and wishful thinking. Furthermore, parents of children with ASD experienced multiple conflicts, including parental distress, challenging child behaviors, dysfunctional parent- child interactions, and self-blame.

### Comparisons of PSI-SF and CSQ between groups

Results are shown in Table 2. Significant differences were observed (effect size *ω*^2^ = 0.74) across all subscales of the Parental Stress Index—Short Form, with total parental stress being highest in the ASD group than in the ADHD and NTD groups. On the parental distress subscale, parents of children with ASD reported significantly higher levels of distress than those in the ADHD and NTD groups, who also differed from each other (*ω*^2^ = 0.26). For dysfunctional parent–child interaction, scores were significantly higher in the ASD group, while the ADHD and NTD groups showed similar and lower levels (*ω*^2^ = 0.41). On the difficult child subscale, all three groups differed significantly, with the highest scores in the ASD group, followed by ADHD, and the lowest in the NTD group (*ω*^2^ = 0.65).

**Table 2:** PSI-SF and CSQ domains reported for parents of children with NTD, ADHD, and ASD.

Variable	NTD (*n* = 79)	ADHD (*n* = 65)	ASD (*n* = 67)	ES
				(*ω*^2^/Cramer’s *V*)
Parental Stress Index	–			
Parental distress^a^	21.73 ± 6.13c	29.31 ± 9.49a	34.82 ± 11.41b	0.26
Dysfunctional parent-child interaction^a^	21.63 ± 6.47c	30.55 ± 11.45a	39.07 ± 7.63b	0.41
Difficult child^a^	24.3 ± 5.94c	28.22 ± 4.8a	42.85 ± 6.73b	0.65
Total stress level^a^	67.67 ± 11.95c	88.08 ± 1.66a	116.75 ± 16.74b	0.74
Degree of parental stress^c^				0.98
Low (<59)	19 (24.05%)	0	0	
Moderate (60–82)	58 (73.41%)	0	0	
High (83–90)	2 (2.53%)	65 (100%)	0	
Clinically relevant (>91)	0	0	67 (100%)	
Coping Styles Questionnaire				
Problem focused				
Problem solving (ratio)^a^	0.45 ± 0.1a	0.22 ± 0.06b	0.22 ± 0.05b	0.67
Seeking social support (ratio)^b^	0.36 ± 0.09a	0.22 ± 0.1b	0.19 ± 0.09b	0.39
Emotion focused				
Self-blame (ratio)^a^	0.05 ± 0.07c	0.14 ± 0.08a	0.33 ± 0.12b	0.61
Avoidance (ratio)^a^	0.09 ± 0.07a	0.15 ± 0.11b	0.14 ± 0.08b	0.08
Wishful thinking (ratio)^a^	0.04 ± 0.05a	0.27 ± 0.13b	0.11 ± 0.06c	0.56
Coping style^c^				0.95
Emotion-focused	4 (5.06%)	65 (100%)	67 (100%)	
Problem-focused	75 (94.93%)	0	0	

ES, effect size.

Different letters indicate statistically significant differences between groups during multiple comparisons. Means and standard deviations (M ± SD) for quantitative variables, and frequencies and percentages (*n*, %) for categorical variables of PSI-SF and CSQ domains, reported separately for parents of children with NTD, ADHD, and ASD.

^a^ One-way ANOVA.

^b^ Welch’s ANOVA

^c^ Chi-square test.

Stress severity categories showed a clear distinction between groups (Cramer's *V* = 0.98): all parents in the ADHD group were classified in the “high” stress range (83–90), whereas all parents in the ASD group were in the “clinically relevant” category (>91). In contrast, most NTD group parents fell within the “moderate” range (73.41%), with only a small proportion exhibiting high stress (2.53%) and none reaching clinical levels. Coping styles also varied notably across groups. Parents in the NTD group reported greater use of problem-focused coping, with significantly higher scores in problem-solving (*M* = 0.45) and seeking social support (*M* = 0.36), compared to both ADHD and ASD groups, which showed similar and lower levels in these strategies (*ω*^2^ = 0.67 and 0.39, respectively). Conversely, emotion-focused strategies were more prevalent among the clinical groups. Self-blame was significantly higher in the ASD group, followed by the ADHD group, with the lowest levels in the NTD group (*ω*^2^ = 0.61). Avoidance was more frequent in both clinical groups than in the NTD group (*ω*^2^ = 0.08). For wishful thinking, the ADHD group showed the highest use, followed by ASD and then NTD (*ω*^2^ = 0.56). Finally, analysis of overall coping style showed a stark contrast: nearly all NTD parents (94.93%) relied on a problem-focused coping style. In comparison, 100% of parents in both the ADHD and ASD groups used emotion-focused coping strategies (Cramer's *V* = 0.95).

### Emotion-focused coping style as a risk factor for parental stress

**Figure 3:**
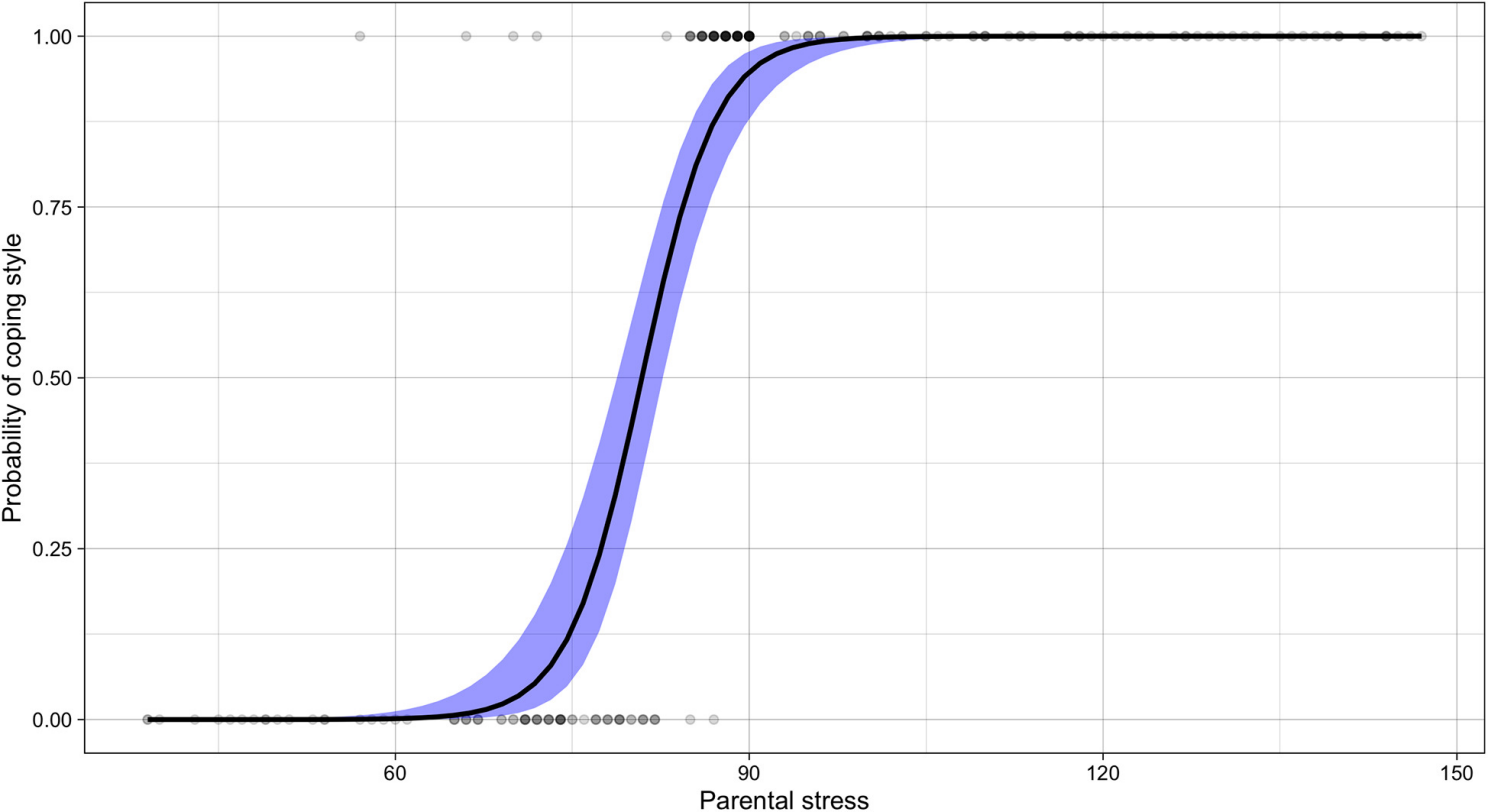
Sigmoid curve of the relationship between parental stress level and coping style. A sigmoid curve of predicted probabilities illustrates the relationship between parental stress (*x*-axis) and coping style (*y*-axis). The logistic function models the probability of using an emotion-focused coping style (coded as 1), with values above 0.50 indicating a greater likelihood of emotion-focused coping, and values below 0.50 indicating a tendency toward problem-focused coping (coded as 0). The steepest segment of the curve represents the stress levels at which the coping style is most likely to shift. Arrows indicate the direction of increasing probability toward emotion-focused coping as parental stress intensifies, highlighting the transition point where coping strategies may begin to change in response to higher stress level.

An emotion-focused style emerged as a risk factor for parental stress (OR = 1.37, 95% CI = 1.25–1.51, *z* = 6.48), indicating that using an emotion-focused strategy increased the odds of higher parental stress by 37%, as illustrated in Figure 3. The model explained 77.84% and 70.1% of the discrimination and resolution, respectively, according to the Tjur and McFadden coefficients.

## Discussion

The primary objective of this study was to evaluate the stress levels and coping strategies of parents of children with ASD, ADHD, and NTD, and to examine the differences between these groups. The findings of the study indicated that parents of children with ASD exhibited higher levels of stress in comparison to parents of children with ADHD and NTD. Furthermore, the study revealed that parents of children with ASD and ADHD were more inclined to utilize negative emotion-focused coping strategies. In contrast, parents of children with NTD were more likely to employ positive problem-focused coping strategies. These findings suggest a significant correlation between the stress levels experienced by parents and the coping strategies they report using, underscoring a discernible shift in coping strategies as stress levels escalate. It is essential to acknowledge that such coping patterns may not solely reflect individual choice but could also be influenced by limited access to external resources and inadequate structural support systems. Parents' use of specific coping strategies is not merely a matter of personal preference but often reflects the structural limitations they face. Emotion-focused strategies, while sometimes viewed as less adaptive, may emerge when parents lack adequate social, psychological, or institutional support to engage in problem-solving coping (10, 39).

Research has documented elevated rates of clinically significant stress in parents of children with ASD and ADHD. In parents of children with ASD, the figures range from 85% to 90.1% (5, 16, 39–41), while in parents of children with ADHD, the range is 78% to 88% (42, 43). Conversely, parents of children with NTD exhibit markedly lower prevalences (15.4%–22%) (40, 42). These results are consistent with those reported in this study.

As posited by Marcinechová et al. (16) and Pardo-Salamanca et al. (3), the heightened levels of stress experienced by the parents of these children can be attributed to the multifaceted behavioral and developmental demands intrinsic to their conditions, as limited social support, uncertainty surrounding their children's prospects, and the arduous nature of specialized care. The challenges faced by these parents are further compounded by difficulties in accessing suitable services, highlighting systemic gaps prevalent in both groups (17, 18, 44, 45, 46). However, as Piscitello et al. (11) observe, parents of children diagnosed with ADHD may have comparatively greater access to resources and support than parents of children diagnosed with ASD, highlighting the stressors parents encounter, which are directly influenced by their child's specific neurodevelopmental disorder.

Our findings demonstrate a high prevalence of negative emotion-focused coping strategies, particularly among parents of children with ASD, which is consistent with the results of Ntre et al. (17), who suggest that the severity of children's symptoms and the caregiving demands placed on parents often limit their ability to use effective coping strategies. In contrast, Sartor et al. (18) suggest that positive coping strategies are not directly linked to high stress levels. Our study revealed a significant correlation between negative coping strategies and elevated stress levels. This association may be indicative of the potential role of negative coping strategies in parental stress experiences. The PCA results revealed a noteworthy visual separation between parents of autistic children and those of children with ADHD, suggesting distinct stress-related response patterns between these groups. This differentiation may reflect unique challenges associated with each condition—such as sensory sensitivities and rigidity in autism vs. impulsivity and emotional dysregulation in ADHD—that contribute to qualitatively different parenting experiences. These patterns align with previous literature indicating differential parental burdens depending on the child's diagnosis. Although exploratory, the PCA complements our regression and correlation findings by highlighting the multidimensional nature of parenting stress and supporting the value of diagnosis-specific support strategies.

During the first three years of life, parenting stress within the family system has a significant impact on the child's emotional and behavioral development, and this influence remains relatively stable throughout the preschool years.

However, this relationship is not strictly unidirectional. A growing body of evidence highlights the bidirectional nature of this interaction, where child behavioral problems can also intensify parental stress, creating a cycle that affects overall family functioning and mental health (7, 11). When parents employ positive coping strategies and receive adequate social or psychological support, this loop can be interrupted, leading to more nurturing and supportive family environments (4–6). Such environments, in turn, foster improved emotional regulation, resilience, and social competence in children, especially during these pivotal developmental stages (13, 15, 47–49).

It is noteworthy that parents encountering more severe and intricate challenges may be more inclined to depend on emotion-focused coping mechanisms to regulate their emotional responses. Consequently, greater attention should be directed towards parents of children with more significant difficulties, as they may require additional support and tailored interventions. The efficacy of coping strategies, particularly those rooted in self-efficacy and problem-solving, is crucial in reducing stress and fostering positive family dynamics (4, 50). Research has demonstrated that enhancing parental self-efficacy can lead to improvements in understanding of the child's needs, thereby empowering parents to confront challenges with greater confidence (18, 40, 45, 48). Consequently, this fosters the development of a more nurturing home environment that supports the child's emotional well-being.

Positive strategies are crucial for enhancing resilience, resource mobilization, and family adaptability. However, in families of children with ASD, these strategies are often disrupted due to the challenges posed by difficulties in negating social environments and establishing supportive networks, which, in turn, may limit their effectiveness, highlighting the need to identify protective factors that can mitigate the onset or impact of stress (11, 12, 15, 16). While parents who engage in positive coping strategies tend to experience greater well-being, many of the factors identified thus far are relatively stable and resistant to change, which reduces the potential for transformative interventions. This underscores the importance of investigating modifiable factors that can empower parents to adopt positive coping strategies and enhance their ability to manage stress (14, 17).

The present study suggests a significant relationship between the high levels of stress experienced by parents of children with ASD and ADHD and the emotion-focused coping strategies they use. It is therefore vital to emphasize the importance of tailoring support programs to the specific needs of parents, ensuring that they have access to appropriate resources and services from the time their child is diagnosed. This will enable the effective addressing of their specific needs and reduce long-term stress (16, 17). The enhancement of parental resilience and the promotion of positive coping strategies, such as seeking social support, have been demonstrated to serve as a protective buffer against everyday stressors and to reduce the risk of depression. The provision of psychological support to parents has been shown to lead to improvements in parental mental health and family well-being, while concurrently having a favorable impact on the developmental outcomes of their children (8, 14). Consequently, targeted interventions that prioritize the well-being of both parents and children are imperative to cultivate healthier and more resilient family dynamics (10–12, 15, 18, 45).

It is imperative that support programs concentrate on acceptance and social support for parents of children with ASD (7, 9, 40, 44, 51) and behavior management strategies for parents of children with ADHD (4, 45–49, 52). While resolving behavioral issues in children may alleviate some parental stress, broader stress management and emotional regulation strategies are necessary to mitigate the adverse effects of chronic stress and promote emotional well-being for these parents.

The findings of this study must be interpreted considering the Mexican sociocultural and structural context, which may shape parental experiences differently from those in higher-income or more resource-rich settings. In Mexico, limited public access to specialized neurodevelopmental services, regional disparities in healthcare infrastructure, and persistent stigma surrounding mental and developmental conditions may amplify parental stress and restrict the use of adaptive coping strategies. These conditions contrast with countries where formal support networks, early intervention programs, and broad awareness initiatives are more established. This may help explain the predominance of emotion-focused coping observed in our sample. The predominance of maternal respondents also reflects cultural norms in Mexico, where caregiving roles are traditionally assigned to women. These contextual differences highlight the need for culturally responsive interventions and reinforce the relevance of the current study in expanding the global literature on parenting stress and coping across diverse socio-political settings (19, 20).

Based on the findings of this study, targeted interventions should prioritize the development of adaptive coping strategies and core cognitive-behavioral competencies associated with effective stress management. These may include training in problem-solving, thought regulation, proactive planning, and relaxation techniques. Additionally, incorporating mindfulness and acceptance-based components could enhance parents' ability to maintain emotional balance and recognize positive experiences in the face of stress. Such integrative interventions may be especially beneficial for parents of children with neurodevelopmental conditions, helping to strengthen resilience and promote well-being (53).

Despite the paucity of research examining the underlying factors contributing to both parental stress and coping, and the limited focus on the differential impact of child neurodevelopmental problems across clinical groups, this study provides valuable insights as it sheds light on the complexities of parenting within three distinct clinical groups, focusing on the nuanced interplay between stressors and coping mechanisms.

### Strengths, limitations and future directions

This study addresses the limitations of previous research (50, 54) by including a larger and more diverse sample from three educational institutions, which represent a broad range of socio-economic and cultural strata, thereby enhancing the generalizability of the findings. The use of validated instruments, such as the CEA and PSI-SF, is instrumental in ensuring the reliability and validity of the study. By focusing on parents of preschool children aged 3–5 years, a critical developmental stage for neurodevelopmental disorders, it fills a gap in the literature, offering valuable insights into parental stress and coping strategies. To the best of our knowledge, no previous comparative descriptive studies have examined the relationship between stress and coping strategies across these clinical groups in the Mexican context.

It is imperative to interpret the significant correlations between parental stress and coping strategies with caution, considering the possibility of shared method variance. Given that both constructs were measured using self-report instruments completed by the same respondents, common method bias may have contributed to the strength of the observed associations. This limitation is well-documented in psychological and behavioral research that relies on single-source data and may lead to inflated estimates of correlation due to respondents' consistent response tendencies or affective states at the time of survey completion (55). Future studies should consider incorporating multi-informant approaches or objective measures to enhance the validity of their findings, thereby mitigating the influence of method-related artifacts and enhancing the robustness of these associations. In the binomial logistic regression analysis, emotion-focused coping emerged as a significant risk factor associated with higher levels of parental stress, increasing the odds of being in the high-stress group. However, given the cross-sectional design of the study, this term is used in a statistical sense to reflect the strength of the association rather than to imply a causal relationship. These findings highlight that parents who tend to rely on emotion-focused coping strategies may be more likely to report elevated stress levels, though the directionality of this relationship remains unclear in our study.

The study's reliance on a single source of information, primarily mothers, restricts the range of perspectives and the extent of the findings concerning potential disparities in the coping experiences of fathers and mothers, as previously noted in other reviews (56, 57). This underrepresentation was not intentional, but rather the result of mothers' greater willingness to participate in studies related to their children's mental health and well-being, a trend also reported in the literature (10, 48, 58). Social stigma also posed a barrier to broader family participation. While surpassing the threshold of 100 participants strengthens the statistical power and reliability of drawing reliable conclusions, as highlighted by Yorke et al. (59), the cross-sectional design limits the ability to establish causal relationships between coping strategies and stress levels.

Moreover, the study did not account for the type, intensity, or duration of behavioral and pharmacological interventions received by the children, which may significantly influence levels of parental stress. Variations in access to services, adherence to treatment, and the specific therapeutic approaches used (e.g., behavioral therapy, occupational therapy, stimulant medications) across groups could play a key role in shaping parental coping patterns and psychological outcomes. Future research should address these gaps by incorporating more diverse family perspectives—particularly those of fathers—and by examining treatment-related factors within longitudinal designs that enable a deeper understanding of how these variables evolve and interact over time. Similarly, strategies for over-recruitment or targeted sampling of parents should be incorporated to bridge this gap and enrich our understanding of gender differences in parental coping with neurodevelopmental conditions.

Furthermore, it is imperative to examine cultural factors that influence parental stress and coping mechanisms. Such investigations are essential for developing culturally sensitive interventions tailored to the diverse contexts of families. While variables such as the child's diagnosis, core symptoms of the condition, co-occurring behavioral issues, social stigma, and financial strain are relatively stable and less amenable to change, the exploration of additional unexamined factors such as influence of social support networks, cultural factors, parental mental health, economic factors, diagnosis-specific demands and longitudinal changes in family dynamics are warranted. A nuanced understanding of these cultural dimensions is crucial for developing inclusive and practical support programs that cater to the unique needs of families from diverse backgrounds.

Additionally, the neurodivergent status of the participating parents was not assessed in this study. Given that parents of children with ASD or ADHD may themselves present neurodevelopmental traits or conditions, which could influence both stress perception and coping strategies, this represents a relevant variable for future research to consider to obtain a more comprehensive understanding of parental well-being in neurodiverse families.

Although only one participant was excluded due to incomplete data, this decision was necessary to preserve the internal consistency of the analyses. However, even minimal exclusions may limit generalizability, and future studies should consider employing strategies such as multiple imputations or sensitivity analyses to account for missing data while minimizing potential bias.

The data also indicates significant implications for the development of public policy. In the Mexican context, where access to specialized neurodevelopmental services remains limited and social stigma persists, the identification of specific stressors and coping deficits among parents can inform the development of family- centered support programs. Evidence-based policy initiatives aimed at reducing parental stress—through early diagnosis, accessible behavioral interventions, and culturally sensitive psychosocial support—may contribute to improving both parental well-being and child developmental outcomes. Consequently, the present study provides a substantial empirical foundation to support the development and implementation of targeted public health strategies at both local and national levels.

## Conclusion

This study underscores the heightened levels of stress experienced by parents of children diagnosed with ASD and ADHD, who demonstrate a greater propensity for emotion-focused coping strategies. Conversely, parents of children with NTD are more inclined to utilize problem-focused strategies. These findings suggest the need for further exploration of interventions that enhance coping mechanisms and stress management in parents of children with developmental disorders. Importantly, such interventions have the potential to improve both parental well-being and children with ASD and ADHD. The use of emotion-focused strategies should not be interpreted as a mere personal preference, but rather as a response shaped by limited access to structural, emotional, and social support. Interventions, therefore, should not focus solely on modifying coping styles but also on improving systemic support that empower families and promote both parental well-being and more supportive environments for children with ASD and ADHD.

## Data Availability

The raw data supporting the conclusions of this article will be made available by the authors, without undue reservation.
